# SG-APSIC1116: Assessing COVID-19 symptoms in infected healthcare workers in Vietnam

**DOI:** 10.1017/ash.2023.27

**Published:** 2023-03-16

**Authors:** Dinh Thi Thu Huong, Le Nguyen Minh Hoa, Bui Thi Thuy, Nguyen Van Kinh, Pham Ngoc Thach

**Affiliations:** National Hospital for Tropical Diseases, Hanoi, Vietnam; National Hospital for Tropical Diseases, Hanoi, Vietnam; National Hospital for Tropical Diseases, Hanoi, Vietnam; National Hospital for Tropical Diseases, Hanoi, Vietnam; National Hospital for Tropical Diseases, Hanoi, Vietnam

## Abstract

**Objectives:** In early 2021, when the COVID-19 vaccine was scarce in Vietnam, healthcare workers (HCWs) were prioritized for vaccination due to high risk of occupational exposure. However, there is some COVID-19 vaccine hesitancy within HCW communities. Assessing COVID-19 severity among vaccinated and nonvaccinated HCWs would contribute essential information to assure people of vaccine effectiveness and reduce vaccine hesitancy. **Methods:** We conducted a descriptive cross-sectional study at the National Hospital for Tropical Diseases in Hanoi, Vietnam, from May to June 2021. Clinical and epidemiological data from HCWs with positive polymerase chain reaction (PCR) results were collected. The severity of symptoms were classified according to Vietnam Ministry of Health guideline (Decision no. 3416 issued  July 14, 2021) into 5 categories: asymptomatic, mild, moderate, severe, and critical conditions **Results:** Overall, 25 HCWs qualified for this study (14 women and 11 men), with a median age of 31 years. Among them, 3 HCWs were infected due to community exposure, and the rest were infected due to occupational exposure. Also, 3 HCWs received the Astra Zeneca vaccine before being infected with SARS-CoV-2 (one fully vaccinated with 2 doses and the other 2 had had the first dose). Categorized by the severity of infection, 28% were asymptomatic, 44% had mild symptoms, 20% had moderate symptoms, and 8% experienced severe symptoms. All 3 vaccinated HCWs showed only mild symptoms. Cough and sore throat were the main symptoms recorded (60%), followed by fever (56%). Blood test results did not show significant differences between the severe and mild COVID-19 groups. **Conclusions:** COVID-19 vaccination reduced the severity of COVID-19 in this small sample of HCWs. Full COVID-19 vaccination is strongly recommended for HCWs to reduce the spread of COVID-19 and to limit the number of cases with severe disease.

